# Supplementary material for: Using novel internet-based sources for Population Size Estimation of Gay, bisexual, and other cisgender men who have sex with men
Source: medRxiv. 2026 Sep 16:2026.09.14.26363087. Preprint. [Version 1] doi: 10.64898/2026.09.14.26363087 (PMC13596503; doi:10.64898/2026.09.14.26363087)
Supplement: Supplementary file 1 [file NIHPP2026.09.14.26363087v1-supplement-1.pdf]

Supplement Table 1 Sensitivity analysis comparing national Google Trends–derived GBMSM population size estimates using alternative search terms with UNAIDS estimates

| Country | "Gay sex"/"sex" GTPSE, n (%) | "Anal sex"/"sex" GTPSE, n (%) | UNAIDS PSE, 2025 |
|---|---|---|---|
| Senegal | 67,253 (1.44) | 1,955 (0.04) | 58,100 |
| Ghana | 99,471 (1.17) | 78,003 (0.92) | 59,800 |
| Mozambique | 23,038 (0.30) | 47,995 (0.62) | 17,400 |
| Indonesia | 1,335,478 (1.79) | 752,382 (1.01) | 873,400 |

**Abbreviations:** GBMSM, gay, bisexual, and other cisgender men who have sex with men; GT, Google Trends; GTPSE, Google Trends population size estimate; PSE, population size estimate; UNAIDS, Joint United Nations Programme on HIV/AIDS.
